# Automated differentiation of mixed populations of free-flying female mosquitoes under semi-field conditions

**DOI:** 10.1038/s41598-024-54233-3

**Published:** 2024-02-12

**Authors:** Brian J. Johnson, Michael Weber, Hasan Mohammad Al-Amin, Martin Geier, Gregor J. Devine

**Affiliations:** 1 Mosquito Control Laboratory, QIMR Berghofer Medical Research Institute, Brisbane, QLD 4006 Australia; 2 Biogents AG, Weissenburgstr. 22, 93055 Regensburg, Germany

**Keywords:** *Aedes aegypti*, *Anopheles stephensi*, Smart trap, Optoacoustic, Mosquito surveillance, Entomology, Engineering

## Abstract

Great advances in automated identification systems, or ‘smart traps’, that differentiate insect species have been made in recent years, yet demonstrations of field-ready devices under free-flight conditions remain rare. Here, we describe the results of mixed-species identification of female mosquitoes using an advanced optoacoustic smart trap design under free-flying conditions. Point-of-capture classification was assessed using mixed populations of congeneric (*Aedes albopictus* and *Aedes aegypti*) and non-congeneric (*Ae. aegypti* and *Anopheles stephensi*) container-inhabiting species of medical importance. *Culex quinquefasciatus*, also common in container habitats, was included as a third species in all assessments. At the aggregate level, mixed collections of non-congeneric species (*Ae. aegypti*, *Cx. quinquefasciatus*, and *An. stephensi*) could be classified at accuracies exceeding 90% (% error = 3.7–7.1%). Conversely, error rates increased when analysing individual replicates (mean % error = 48.6; 95% CI 8.1–68.6) representative of daily trap captures and at the aggregate level when *Ae. albopictus* was released in the presence of *Ae. aegypti* and *Cx. quinquefasciatus* (% error = 7.8–31.2%). These findings highlight the many challenges yet to be overcome but also the potential operational utility of optoacoustic surveillance in low diversity settings typical of urban environments.

## Introduction

Accurate and timely mosquito surveillance is crucial for improving the effectiveness and evaluation of vector control measures. Unfortunately, traditional surveillance methods are often hindered by a lack of expert human resources and logistical difficulties^1,2^. There is clearly tremendous utility in the development of robust and reliable automated trapping systems, or “smart” traps. By differentiating between insect species and transmitting counts remotely and in real-time^3^, these traps offer a robust and reliable solution for mosquito surveillance^4–6^.

Most smart trap prototypes rely on either image or acoustic data acquisition, but it is the optoacoustic capture of mosquito wingbeat frequencies (WBF) that has historically received greatest attention^7–11^. The greater focus of WBF is likely due to the inherent difficulties in remotely imaging insects to sufficiently control for variations in color, detail, focus, and angle^12^. These variations pose a significant challenge when it comes to picturing mosquitoes in a way that reliably reveals their distinguishing morphological features. In response, image-based surveillance has been most widely adopted by citizen science campaigns wherein publicly captured images of mosquitoes are sent to a central repository for analysis by trained medical entomologists or taxonomists^13–15^. Such campaigns are great public engagement tools with the potential to track broad species distributions and exotic incursions, but they are not yet suitable for traditional surveillance operations. However, substantial progress has been made recently in overcoming historic limitations^16–20^, and it is expected that the number of field-tested photographic smart traps^21^ will increase significantly in the coming years.

Despite the heightened attention that optoacoustic surveillance has received, it is important to acknowledge that reliance on WBF as a diagnostic marker for species separation presents its own unique challenges. While WBF may differ markedly between species due to sexual selection^22–25^, the range of female WBF is narrow and variations may occur in response to environmental, physiological, and behavioral factors^26–30^. Resulting frequency overlap amongst closely and distantly related species^7,8^ can greatly impede accurate species differentiation^7,8^. While simple logical extensions to classification algorithms such as the time and place of capture may help to reduce potential confusion between species, some confusion is likely to remain. In spite of these challenges, WBF-based classification remains promising when there is a clear distinction between the genera being observed and when the number of species being identified is relatively small.

Here, we describe the development, performance and limitations of an innovative optoacoustic smart trap design that enabled us to reliably differentiate congeneric and non-congeneric species under free-flight conditions. We focus on the differentiation of medically and economically important mosquito species inhabiting low-diversity urban environments^31,32^. Critically, each release scenario is designed to represent a current real-world surveillance challenge, including:

- *Scenario 1* Differentiation of the globally invasive, container-breeding mosquitoes *Aedes albopictus* and *Aedes aegypti* in the presence of *Culex quinquefasciatus*. Improving the differentiation of these species is essential for exotic species monitoring, particularly in first ports^33,34^, and public health surveillance. *Ae. aegypti* and *Ae. albopictus* are globally invasive and are competent vectors of dengue, chikungunya, and other important diseases^35^ and both are commonly collected together with *Cx. quinquefasciatus*^36–40^.
- *Scenario 2* Differentiation of *Anopheles stephensi* and *Ae aegypti* in the presence of *Cx. quinquefasciatus.* Discriminating these species is critical to improving our understanding of the relative abundance, distribution, and host-seeking activity of the introduced malaria vector *An. stephensi*^41^ in urban environments in the Horn of Africa. *An. stephensi* is currently known to share larval habitats with *Ae. aegypti* in Africa^42^ and it is collected with both *Ae. aegypti* and *Cx. quinquefasciatus*, a widely distributed species in Africa^43^, elsewhere in its range^44^.

## Results

### Wingbeat frequency datasets

**Figure 1:**
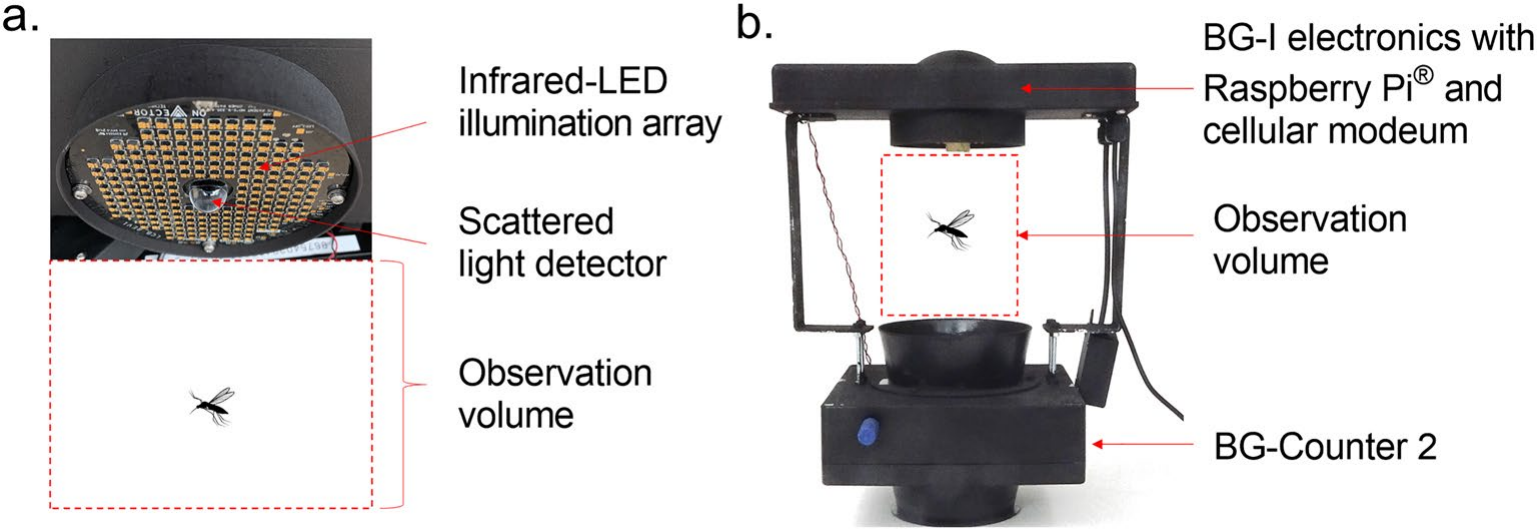
Overview of the tested BG-I optoacoustic trap design. (a) The infrared-LED sensor array of the BG-I trap system is positioned 20 cm above an integrated BG-Counter 2® (Biogents AG, Regensburg, Germany) unit. In the space between the IR-LED array and the BG-Counter trap funnel (the ‘observation volume’), mosquitoes are in free flight. They reflect and scatter IR light as a superposition of a constant ‘body signal’ and a time-varying wingbeat signal. This signal is recorded continuously using a large-area photodiode, transimpedance amplifier and analog-to-digital converter. The digitized wingbeat recording is stored in a ring buffer. (b) Incorporation of the BG-I electronics and sensor array with the commercially available BG-Counter 2® (Biogents AG, Regensburg, Germany) smart trap system. The BG-Counter 2 provides a trigger signal to the BG-I to retrieve the data from the wingbeat recorder from the preceding 200 ms of flight in the observation volume, marking the point just before the mosquito was captured by the airflow of the suction trap.

**Figure 2:**
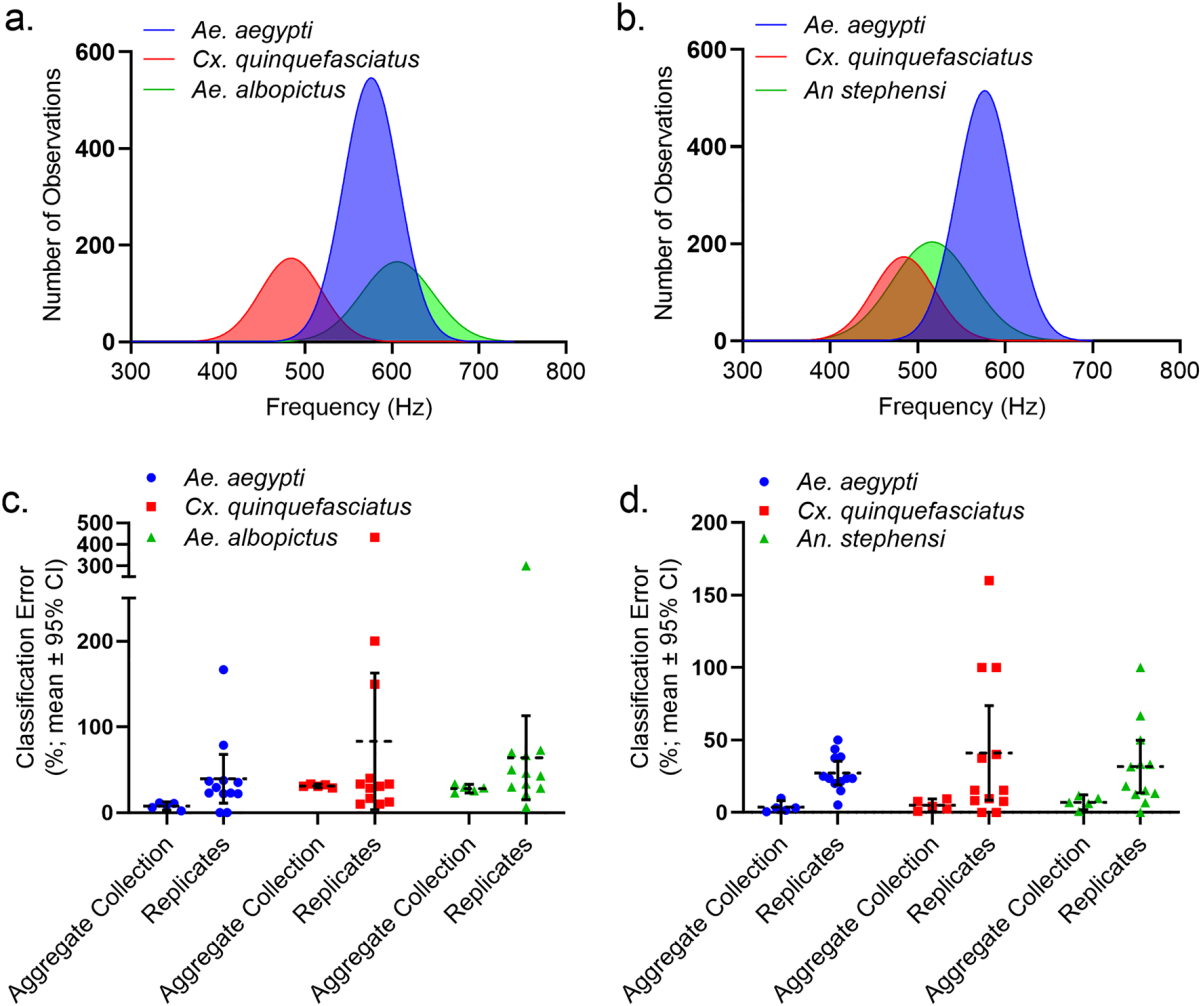
Summary of recorded female wing beat frequency distributions and classification performance of the BG-I trap system for all species released together in each release scenario. (a-b) Wing beat frequency distributions for species released together during release Scenario 1 and Scenario 2, respectively. (c-d) Percent (absolute) error (mean ± 95% CI) of automated (BG-I) species classifications relative to physical trap counts for species released together during release Scenario 1 and Scenario 2, respectively. Percent error observed for each individual replicate is shown as a symbol (n=12).

The developed BG-I trap system (Fig. 1) enabled us to generate substantial WBF datasets (Table 1) for each species tested quickly and without manipulation of insects (i.e., tethering) prior to recording. In total, 5,092 individual recordings were produced from 4,500 released mosquitoes, suggesting an ca. 12% recapture rate. The mean WBFs of mosquitoes assayed spanned 302.6 Hz (Table 1), with the lowest WBF recorded from *Cx. quinquefasciatus* (302.6 Hz) and the highest recorded from *Ae. albopictus* (741.3 Hz). Overlap in WBF distributions, quantified using the overlapping coefficient (OVL)^45^, occurred for all species (Table 2, Fig. 2a,b). Notable overlaps occurred between *Ae. aegypti* and *Ae. albopictus* (OVL = 0.67) and between *Cx. quinquefasciatus* and *An. stephensi* (OVL = 0.68).

**Table 1:** Sample size, signal length, and mean female wingbeat frequency observed for each species recorded in the present study.

Species	Records (N)	Mean wingbeat frequency (Hz)	95% CI of wingbeat frequency	Mean signal duration (ms)	95% CI of duration (ms)
*Ae. aegypti*	2118	573.7	572.2–575.1	101.0	99.1–102.9
*Ae. albopictus*	881	603.9	600.9–607.0	86.02	83.3–88.8
*Cx. quinquefasciatus*	783	483.3	480.7–486.0	119.2	116.3–122.0
*An. stephensi*	1041	512.0	509.2–514.9	121.1	118.4–123.8

**Table 2:** Overlapping coefficients (OVL) among all female wing beat frequency distribution pairs.

Species pair		Overlapping coefficient (OVL)
*Ae. aegypti*	*Ae. albopictus*	0.67
	*Cx. quinquefasciatus*	0.21
	*An. stephensi*	0.43
*Cx. quinquefasciatus*	*Ae. albopictus*	0.15
	*An. stephensi*	0.68
*Ae. albopictus*	*An. stephensi*	0.31

OVL is a measure of similarity between two population distributions. The value of OVL ranges from 0 to 1, where a value 0 indicates that there is no overlap and a value 1 indicating identical distributions.

### Model selection

We found that the tested algorithms, XGBoost and Multilayer Perceptron (MLP), generally performed better in the absence of principal component analysis (PCA), whereas the use of data scaling did not improve general model performance (Tables S1, S2). In both sets of species comparisons, data feature type, i.e., Mel Frequency Cepstral Coefficient (MFCC), Power Spectral Density (PSD), and fundamental frequency, was the only parameter to contribute significantly to classification accuracy (Tables S3, S4). The use of MFCC, in combination with XGBoost algorithm, produced the most accurate classification models, with few exceptions. The use of PSD with XGBoost generally produced more accurate predictions than the use of MFCC for the classification of *Ae. aegypti*, *Ae. albopictus*, and *Cx. quinquefasciatus*, but this model was found to underperform relative to MFCC-based models when classifying replicate data. Based on chosen models, *Ae. aegypti* was predicted to be the identified with the greatest sensitivity, whereas *Ae. albopictus* and *An. stephensi* were predicted to be the identified with the lowest expected sensitivity (Table 3)*.* Final chosen models for each combination of species included the use of MFCC without data scaling or cleaning in combination with the XGBoost classification algorithm.

**Table 3:** Summary of model classification performance (confusion matrix) for each release scenario.

Known	Predicted: scenario 1		
	*Ae. aegypti*	*Ae. albopictus*	*Cx. quinquefasciatus*
*Ae. aegypti*	0.93	0.07	0.00
*Ae. albopictus*	0.50	0.48	0.02
*Cx. quinquefasciatus*	0.02	0.00	0.98

Known	Predicted: scenario 2		
	*Ae. aegypti*	*An. stephensi*	*Cx. quinquefasciatus*
*Ae. aegypti*	0.94	0.06	0.00
*An. stephensi*	0.18	0.74	0.08
*Cx. quinquefasciatus*	0.02	0.06	0.92

Final classification models included the use of MFCC with no data scaling or cleaning in combination with the XGBoost classification algorithm.

### Application of classification models to free-flight data

Both levels of data cleaning, aimed at removing weak or incomplete recordings, resulted in the loss of true observations and the underreporting of trap totals in both release scenarios (Table 4), with significant losses of true observations when full data cleaning was employed for Scenario 1 (*F*_2,33_ = 8.7, *P* < 0.01). The high agreement between raw remote and physical counts indicates a low occurrence of false recordings with the current trap configuration. As a result, raw remote recordings were used for all subsequent analyses.

**Table 4:** Departure of BG-I trap counts from physical trap counts at different levels of data cleaning for each free-flight release scenario.

	No clean		Low clean		Full clean		Physical count (n)	
	Mean (%)	SEM	Mean (%)	SEM	Mean (%)	SEM	Mean	SEM
Scenario 1	0.18	1.99	7.64	2.99	12.10	2.96	28.58	2.11
Scenario 2	1.40	1.30	6.10	1.86	10.65	1.47	36.83	1.70

Physical count represents the mean number of mosquitoes collected per replicate.

At the aggregate level (all collections summed per release scenario), automated classification of all species released in Scenario 2 was nearly accurate, with mean classification accuracy exceeding 90% (% error = 3.74–7.08%; Table 5; Fig. 2c,d). Classification error increased in Scenario 1 (% error = 7.79–31.23%) but was again highly accurate for *Ae. aegypti* (% error = 7.79, 95% CI 3.0–12.56%). In contrast, classification error increased markedly across individual replicates (mean % error = 44.7; 95% CI 30.0–59.5%; Fig. 2c,d) for both release scenarios. Mean species classification error across all replicates ranged from 29.6 to 63.4% (Table 5).

**Table 5:** Percent (%) error of individual species classifications at the replicate and aggregate levels for both release scenarios.

Release scenario 1			
	*Ae. aegypti*	*Cx. quinquefasciatus*	*Ae. albopictus*
Replicate/aggregate Mean	39.57/7.79	83.21/31.23	64.00/28.07
Replicate/aggregate Lower 95% CI	11.10/3.01	3.43/29.11	15.05/23.02
Replicate/aggregate Upper 95% CI	68.04/12.56	162.98/33.35	112.94/33.12

Release scenario 2			
	*Ae. aegypti*	*Cx. quinquefasciatus*	*An. stephensi*
Replicate/aggregate Mean	27.25/3.74	41.15/5.00	31.72/7.08
Replicate/aggregate Lower 95% CI	19.15/0.77	8.54/0.56	13.52/1.86
Replicate/aggregate Upper 95% CI	35.35/8.25	73.36/9.44	49.92/49.92

## Discussion

To our knowledge, we report findings from the first mixed-species test of a field-ready optoacoustic smart trap design under free-flight conditions. While others have achieved automatic classification of mosquito genera^46^, sex^47,48^, or both^49^, none have attempted point-of-capture classification of mixed-species populations of free-flying mosquitoes. The work presented highlights both the challenges and opportunities presented by optoacoustic surveillance. For instance, we estimate that, for aggregate collections, non-congeneric species present in release scenario 2 (i.e., *Ae. aegypti*, *Cx. quinquefasciatus,* and *An. stephensi*) could be quantified with accuracies exceeding 90%. However, error rates increased when analysing individual (replicate) collections in both release scenarios and for aggregate collections containing *Ae. albopictus* in the presence of both *Ae. aegypti* and *Cx. quinquefasciatus*. These findings show the challenges yet to be overcome as well as the potential operational utility of optoacoustic surveillance in low diversity settings typical of urban habitats^50^.

The study importantly introduces a novel optoacoustic smart trap design that increased the depth of acquired optoacoustic signals (signal length = 86–121 ms) relative to existing designs^49,51^. The success of the tested design is attributed to its above-trap sensor placement that allows for prolonged signal acquisition. Acquired signal depth was sufficient for accurate quantification of aggregate collections of mixed-species under free-flight conditions in the presence of significant WBF overlap. The tested design was further found to be robust to false data acquisition, or captures, as supported by the low divergence of physical and remote trap counts across both release scenarios. However, it is important to acknowledge the absence of negative samples, or non-mosquito bycatch, in the present study. Although mosquito bycatch in non-light traps, such as the BG-Sentinel and the tested trap design, is low in comparison to traditional light traps (e.g., CDC-miniature light trap)^52^, bycatch collections can still exceed mosquito captures^53^. Further research is needed to better understand how the presence of different groups of non-target insects may influence classification performance.

The study further presents a rare test of laboratory-trained classification algorithms against small, mixed-species collections typical of daily trap captures. The accuracy of our classification algorithms decreased significantly when classifying low replicate sample sizes relative to larger trap aggregates. This observation is critical since model validation has not been successfully extended beyond large WBF databases containing one or more separately recorded datasets^47,49,54^. The lower accuracies observed across individual replicates suggest that the best approach for mixed-species classification is to aggregate trap collections over time or space. For low density species, such as *Ae. aegypti*^55,56^, this may cause significant delays in analysis in the absence of extended trap networks, which may not be suitable for certain surveillance operations.

It is worth noting that the decrease in model performance when applied to individual replicates relative to trap aggregates was unexpected. Although small datasets tend to cause problems of model overfitting or underfitting in machine learning, these problems typically refer to the initial training of the chosen machine learning algorithm^57,58^. As reasonably large training data sets were employed, discrepancies in classification performance are harder to explain considering the low expected error rates for the majority of species analyzed. Deviations in classification performance may be related to cohort-to-cohort differences (e.g., body size), which may lead to significant over- or underreporting of species counts when analyzing small sample sizes, such as was observed for *Cx. quinquefasciatus* in both release scenarios. Such differences may occur despite the use of single colonies reared under controlled rearing conditions, although these differences are not as large as those observed in field populations^59^. Further research into the performance of classification models against cohorts reared under different environmental and resource conditions is warranted.

The study had further limitations that should be noted. Primary among these is the use of established mosquito colonies reared and released under controlled laboratory settings that likely improved classification accuracy relative to that expected under natural field conditions. This limitation is not unique to this study as it is shared by the majority of previous reports^47–49,54,60^. Mosquito WBF fluctuates in response to environmental, physiological, and behavioral factors^26–30^, and this variability has yet to be adequately accounted for in the field or laboratory by those attempting WBF-based classification. Some of the confusion among species created by this variability may be accounted for by simple logical extensions to classification algorithms, such as the time and place of recording^7^. For instance, separating collections by time of capture may significantly reduce classification errors for the more crepuscular *Cx. quinquefasciatus*^61^ in the presence of day-active mosquitoes like *Ae. aegypti*^62^ and *Ae. albopictus*^63^, but some overlap will persist. Future research should prioritize testing and developing trap designs and classification models under natural field conditions or within semi-field systems situated within the natural environment and exposed to ambient environmental conditions^64,65^.

In conclusion, wing beat-focused smart trap designs find their most obvious application in environments that have low species diversity, such as those that were simulated. However, limitations remain, and an emphasis on field-based studies is needed before integration into traditional surveillance operations. Despite these limitations, the potential operational utility of WBF focused smart traps remains high.

## Methods

### Trap and sensor design

The BG-I trap system (Fig. 1) consists of a bespoke infrared-LED array (192 × SFH-4641-Z, 940 nm, ams-OSRAM AG, Munich, Germany) with imbedded light sensor (Hamamatsu Photonics K.K., Shizuoka, Japan) mounted 20 cm above a BG-Counter 2® (Biogents AG, Regensburg, Germany). The IR-LED and light sensor array (10 cm in diameter) is supported and operated by a Raspberry Pi 3 (Model B, Raspberry Pi Ltd, Cambridge, UK) and SAMD21G microprocessor (Atmel® Corporation, San Jose, CA, USA). Suction is provided by a 12-V (3.6 W) fan positioned 30 cm below the BG-Counter 2 funnel and at a 90-degree angle from the funnel opening to reduce background light scatter.

During operation, the BG-I system continuously measures the intensity of reflected light from the observation volume, i.e., the distance between the sensor array and trap funnel. An individual observation is initiated by the registration of a mosquito capture by the BG-Counter 2 attached to the trap opening. Once a mosquito is registered, the BG-Counter 2 provides a simultaneous trigger signal to the BG-I recorder to retrieve the data collected from the preceding 200 ms, or just before capture. This data corresponds to the small portion of the light scattered and reflected by the insect during capture and transit through the observation volume. The light sensor, or photodiode, output is then amplified using proprietary electronics and then digitized by the 12-bit Analog-to-Digital Converter on the SAMD21G at the rate of 8 kHz. The digitized signal is transmitted to a Raspberry Pi for intermediate storage and transmission to a cloud server. The signals are converted to .wav files prior to analysis.

### Mosquito rearing

A wingbeat database was constructed for each species from established laboratory colonies. All colonies were maintained in environmental chambers at standard conditions (27.0 ± 0.5 °C, 80.0 ± 5.0% RH, and 12:12 (L:D) h photo regime). In general, larvae were reared in 45 cm × 32 cm × 5.5 cm pans containing 1.0 L of reverse osmosis purified water. *Aedes* sp. and *Cx. quinquefasciatus* larvae were reared on a diet of Tetramin fish food (Tetra, Melle, Germany). *Anopheles stephensi* were fed on a standardized diet of Vipan and Micron Nature fry foods (Sera®, Heinsberg, Germany). Emergent adults were placed in 30 × 30 × 30 cm mesh cages (BugDorm, Taichung, Taiwan) and provided 10% sucrose solution ad libitum. Adults were aged 5–7 days at the time of recording.

### Data collection and trap operation

Training datasets were generated for each species in single-species, large cohort capture experiments. A minimum of 3–5 cohorts (releases) reared at different times and from different egg stock were used for each species. Cohort sizes varied from 50 to 300 individuals, depending on adult availability. Adults were released into a 1.5 × 2.0 × 1 m mosquito mesh tent containing the BG-I trap station. CO_2_ was supplied to the trap station from a 6 kg cylinder at a rate of 300 mL/min. Released mosquitoes were captured for a period of 24 h or until no free-flying mosquitoes could be observed in the tent. No collection bag was attached to the end of the trap body to allow for the possible re-capture of mosquitoes. Training datasets were generated for females only.

### Data processing

The BG-I signal was analysed and processed using the librosa^66^, scipy^67^ and peakutils^68^ Python libraries. Three different data feature classes where generated from the BG-I signal, including Mel Frequency Cepstral Coefficients (MFCC)^69^, Power Spectral Density (PSD)^70^, and Fundamental frequency^46^. MFCC is derived from the Fourier transformation of the signal and is a representation of the short-term power spectrum of a sound. PSD describes how the power of a signal is distributed over frequency^70^. Fundamental frequency is defined as the lowest frequency of a periodic waveform and is equivalent to the fundamental WBF. All three feature classes have been used in previous optoacoustic studies^3,46,71,72^. In general, fundamental and harmonic frequencies were determined by harmonic analysis of the Fourier series which also enabled us to reliably identify PSD peaks based on multiples of the fundamental frequency estimate.

After initial signal processing, feature data was preprocessed in two ways prior to model training. First, various levels of data scaling, or standardization, were tested by applying different scalers (e.g., standard scaler, normalizer, and robust scaler) to each dataset prior to classification. Second, each data transformation method was tested without and in combination with principal component analysis (PCA). The use of PCA can reduce the number of input variables which can result in a simpler predictive model with better performance^73^.

### Free-flight capture experiments

The ability of the BG-I station to accurately differentiate and record sympatric species was tested under free-flight conditions using two different combinations of species, or release scenarios. The first scenario consisted of the urban, container-inhabiting congeneric species *Ae. aegypti* and *Ae. albopictus* in the presence of the commonly co-collected *Cx. quinquefasciatus.* The second scenario included the non-congeneric species *Ae. aegypti, An. stephensi*, and *Cx. quinquefasciatus*. Twelve releases of each grouping were performed during which captured mosquitoes were collected, counted manually, and results compared to the clustering results of the preferred classification algorithm. During each individual experiment, 20 females of each species in the necessary combinations were released into a mosquito mesh tent (1.5 × 2.0 × 1 m) containing the BG-I trap station. The one exception being the release of 40 females of *Cx. quinquefasciatus* during each replicate. The larger addition of *Cx. quinquefasciatus* was used to ensure adequate captures of this species as our colonized *Cx. quinquefasciatus* population maintains natural crepuscular peaks in host-seeking activity and collections at other times of the day can be low. Captured mosquitoes were collected in 120 min intervals and all remaining mosquitoes removed by aspiration. Only female mosquitoes were released during free-flight experiments. Experiments occurred between the hours of 0900–1900, with the majority of releases occurring between 1200 and 1300. All free-flight experiments occurred under controlled environmental conditions (27.0 ± 0.5 °C, 80.0 ± 5.0% RH, and 12:12 (L:D) h photo regime). A collection bag was attached to the end of the trap body for all free-flight experiments.

### Post-capture analysis

Captured mosquitoes were chilled at − 20 °C for 30 min before being counted and morphologically identified. Manual species counts were then compared to model predictions of BG-I recordings for each capture interval to determine the difference (%) between remote and manual counts and classification error rates. Three levels of post-capture data cleaning were compared to determine which produced the greatest consensus between remote and manual counts. The three levels are (1) no data cleaning (i.e., raw files analysed), (2) low-level data cleaning to remove recordings with low signal power (< 0.2 × 1000), and (3) full data cleaning to remove recordings with low signal power, those with low harmonic detection (< 3 defined harmonics), and those with low-frequency to power ratios (< 1.0). Signal power was calculated in the time domain using the root mean square (RMS) method^74^ or in the frequency domain (including frequency bands) using Parseval’s theorem^75^. The calculation of RMS power and frequency band power ratios allows for the identification of signals that have low power or a large low-frequency content obscuring the true wingbeat signal.

### Model selection, training and validation

During the training and validation phase, we evaluated the performance of a particular model for each release scenario based on classification accuracy as determined from cross-validation using 80–20% data splitting (training-validation sets). Validation was performed using the *scikit*-learn python package^76^. The number of training iterations exceeded 30 in all cases. The two parent algorithms tested included XGBoost, a decision-tree-based ensemble machine learning algorithm that uses a gradient boosting framework^77^, and Multilayer Perceptron (MLP), a class of feedforward artificial neural network^78^. Each parent algorithm was trained at all levels of data scaling and transformation (i.e., PCA) for an individual feature type. A summary of tested models is presented in Table S1.

Performance of each validated model was assessed against experimental replicates as well as experimental aggregates (i.e., the sum of collections per release scenario). Due to the lack of replication of experimental aggregates, variance in model performance and classification accuracy was assessed using multiple validated models trained from randomly sampled subsets of the training data (n = 5, 75% data retention).

### Statistical analyses

Linear mixed-effects models were used to determine the importance of model type, feature type, and data scaler on classification performance. Species was included as a random effect in the models to account for species-level differences in classification accuracy. Analysis of variance was used to determine if the percent difference of physical and BG-I species counts was influenced by the level of data cleaning employed. Prior to analysis, assumptions of normality and homogeneity of variance were determined using the Shapiro-Wilks test^79^ and Bartlett’s test^80^, respectively. Distributions of test results across replicates was found to satisfy assumptions of normality (*W* = 0.87–0.98, *p* = 0.07–0.98) and homogeneity of variance (χ^2^ = 1.39–2.08, *p* = 0.36–0.50). The null hypothesis for all significance tests was one of no difference among the variables, models, and/or parameters being assessed. The absolute departure of automated and physical species counts was calculated as Percent Error using the formula $\% e=\bigg|\frac{\left(p - a\right)}{a}\bigg|\times 100$, in which *a* represents the physical (actual) collection total and *p* represents the predicted collection total for each replicate or aggregate collection being analysed. All statistical analyses were performed using R version 4.04^81^. Data visualization and cleaning, model training, and post-capture analyses were performed using a custom Python-based (Python Software Foundation, version 3.9) data visualization and analysis platform.

### Supplementary Information

Supplementary Information 1.

Supplementary Information 2.

## Data Availability

Data supporting the findings of this study are available in the Supplementary Data 1. The Python code file is available as a Supplementary Data 2. The python code file may also be found at https://github.com/mweber-bg/BGWingbeatAnalyzerCC.
