# Supplementary material for: Automated differentiation of mixed populations of free-flying female mosquitoes under semi-field conditions
Source: Sci Rep. 2024 Feb 12;14:3494. doi: 10.1038/s41598-024-54233-3 (PMC10861447; doi:10.1038/s41598-024-54233-3)
Supplement: Supplementary file 1 — Supplementary Information 1. [file 41598_2024_54233_MOESM1_ESM.docx]

Automated differentiation of mixed populations of free-flying mosquitoes under semi-field conditions

Brian J. Johnson^1, ⸸,*^, Michael Weber^2,⸸^, Hasan Mohammad Al-Amin^1^, Martin Geier^2^, and Gregor J. Devine^1^

^1^Mosquito Control Laboratory, QIMR Berghofer Medical Research Institute, Brisbane, QLD 4006, Australia
^2^Biogents AG, Weissenburgstr. 22, Regensburg 93055, Germany
^*^brian.johnson@qimrberghofer.edu.au
^⸸^these authors contributed equally to this work

**S1 Table**. **Species-level classification accuracy for release scenario 1.** Classification accuracy for *Ae. aegypti, Ae. albopictus*, and *Cx. quinquefasciatus* obtained for each parent algorithm (XGBoost and MLP) across each tested feature type and data scaler. The absence of a data value indicates an instance of failed model convergence.

|  |  | XGBoost | | | w/PCA | | |
| --- | --- | --- | --- | --- | --- | --- | --- |
| Feature | Scaler | *Ae. aegypti* | *Ae. albopictus* | *Cx. quinq.* | *Ae. aegypti* | *Ae. albopictus* | *Cx. quinq.* |
| PSD | No Scaler | 0.92 | 0.57 | 0.98 | 0.90 | 0.30 | 0.89 |
|  | Standard- Scaler | 0.92 | 0.57 | 0.98 | 0.90 | 0.30 | 0.89 |
|  | Normaliser | 0.91 | 0.57 | 0.92 | 0.89 | 0.30 | 0.86 |
|  | Robust- Scaler | 0.92 | 0.57 | 0.98 | 0.90 | 0.30 | 0.89 |
| MFCC | No Scaler | 0.93 | 0.48 | 0.98 | 0.94 | 0.49 | 0.96 |
|  | Standard-Scaler | 0.93 | 0.48 | 0.98 | 0.94 | 0.49 | 0.96 |
|  | Normaliser | 0.92 | 0.49 | 0.90 | 0.94 | 0.39 | 0.95 |
|  | Robust- Scaler | 0.93 | 0.48 | 0.98 | 0.94 | 0.49 | 0.96 |
| Fundamental | No Scaler | 0.88 | 0.32 | 0.82 | 0.88 | 0.32 | 0.82 |
|  | Standard- Scaler | 0.88 | 0.32 | 0.82 | 0.88 | 0.32 | 0.82 |
|  | Normaliser | 0.88 | 0.32 | 0.82 | 0.88 | 0.32 | 0.82 |
|  | Robust- Scaler | 0.88 | 0.32 | 0.82 | 0.88 | 0.32 | 0.82 |
|  |  |  |  |  |  |  |  |
|  |  | MLP (neural net) | | | w/PCA | | |
| Feature | Scaler | *Ae. aegypti* | *Ae. albopictus* | *Cx. quinq.* | *Ae. aegypti* | *Ae. albopictus* | *Cx. quinq.* |
| PSD | No Scaler | - | - | - | - | - | - |
|  | Standard- Scaler | 0.85 | 0.54 | 0.92 | 0.93 | 0.17 | 0.82 |
|  | Normaliser | 0.89 | 0.54 | 0.89 | 0.92 | 0.21 | 0.84 |
|  | Robust- Scaler | 0.84 | 0.54 | 0.90 | 0.92 | 0.24 | 0.82 |
| MFCC | No Scaler | 0.98 | 0.22 | 0.89 | 0.91 | 0.54 | 0.97 |
|  | Standard-Scaler | 0.94 | 0.53 | 0.97 | 0.93 | 0.52 | 0.97 |
|  | Normaliser | 0.94 | 0.41 | 0.91 | 0.94 | 0.43 | 0.95 |
|  | Robust- Scaler | 0.93 | 0.55 | 0.97 | 0.93 | 0.52 | 0.97 |
| Fundamental | No Scaler | - | - | - | 0.91 | 0.24 | 0.85 |
|  | Standard- Scaler | 0.90 | 0.32 | 0.78 | 0.90 | 0.33 | 0.79 |
|  | Normaliser | - | - | - | - | - | - |
|  | Robust- Scaler | 0.90 | 0.33 | 0.8 | 0.9 | 0.33 | 0.79 |

**S2 Table.**  **Species-level classification accuracy for release scenario 2.** Species-level classification accuracy for *Ae. aegypti, Cx. quinquefasciatus* and *An. stephensi* obtained for each parent algorithm (XGBoost and MLP) across each tested feature type and data scaler. The absence of a data value indicates an instance of failed model convergence.

|  |  | XGBoost | | | w/PCA | | |
| --- | --- | --- | --- | --- | --- | --- | --- |
| Feature | Scaler | *Ae. aegypti* | *An. stephensi* | *Cx. quinq.* | *Ae. aegypti* | *An. stephensi* | *Cx. quinq.* |
| PSD | No Scaler | 0.91 | 0.78 | 0.90 | 0.87 | 0.42 | 0.6 |
|  | Standard- Scaler | 0.91 | 0.78 | 0.90 | 0.87 | 0.42 | 0.6 |
|  | Normaliser | 0.90 | 0.73 | 0.71 | 0.89 | 0.41 | 0.63 |
|  | Robust- Scaler | 0.91 | 0.78 | 0.90 | 0.87 | 0.42 | 0.6 |
| MFCC | No Scaler | 0.94 | 0.74 | 0.92 | 0.91 | 0.67 | 0.87 |
|  | Standard-Scaler | 0.94 | 0.74 | 0.92 | 0.91 | 0.67 | 0.87 |
|  | Normaliser | 0.92 | 0.62 | 0.70 | 0.88 | 0.56 | 0.74 |
|  | Robust- Scaler | 0.93 | 0.79 | 0.90 | 0.91 | 0.67 | 0.87 |
| Fundamental | No Scaler | 0.85 | 0.35 | 0.60 | 0.86 | 0.35 | 0.61 |
|  | Standard- Scaler | 0.85 | 0.35 | 0.60 | 0.86 | 0.35 | 0.61 |
|  | Normaliser | - | - | - | 0.85 | 0.77 | 0.91 |
|  | Robust- Scaler | 0.85 | 0.35 | 0.60 | 0.86 | 0.35 | 0.61 |
|  |  |  |  |  |  |  |  |
|  |  | MLP (neural net) | | | w/PCA | | |
| Feature | Scaler | *Ae. aegypti* | *An. stephensi* | *Cx. quinq.* | *Ae. aegypti* | *An. stephensi* | *Cx. quinq.* |
| PSD | No Scaler | - | - | - | - | - | - |
|  | Standard- Scaler | 0.87 | 0.69 | 0.83 | 0.89 | 0.38 | 0.57 |
|  | Normaliser | 0.90 | 0.75 | 0.62 | 0.9 | 0.42 | 0.56 |
|  | Robust- Scaler | 0.89 | 0.66 | 0.80 | 0.89 | 0.41 | 0.61 |
| MFCC | No Scaler | 0.90 | 0.80 | 0.50 | 0.92 | 0.62 | 0.86 |
|  | Standard-Scaler | 0.92 | 0.82 | 0.95 | 0.9 | 0.72 | 0.91 |
|  | Normaliser | 0.91 | 0.63 | 0.60 | 0.89 | 0.63 | 0.72 |
|  | Robust- Scaler | 0.92 | 0.82 | 0.95 | 0.9 | 0.72 | 0.92 |
| Fundamental | No Scaler | - | - | - | 0.87 | 0.4 | 0.56 |
|  | Standard- Scaler | 0.88 | 0.40 | 0.60 | 0.88 | 0.34 | 0.64 |
|  | Normaliser | - | - | - | 0.85 | 0.77 | 0.91 |
|  | Robust- Scaler | 0.89 | 0.38 | 0.57 | 0.89 | 0.37 | 0.6 |

**S3 Table. Summary of linear mixed-effects models.** Asterisk (*) indicates p-value smaller than 0.05 (p<0.05).

| Classification of *Ae. aegypti*, *Ae. albopictus*, and *Cx. quinquefasciatus* | | | | | |
| --- | --- | --- | --- | --- | --- |
| Variable | Estimate | SE | df | t.ratio | p.value |
| (Intercept) | 0.67 | 0.14 | 118 | 4.72 | <0.001* |
| Feature: MFCC | 0.12 | 0.01 | 118 | 8.26 | <0.001* |
| Feature: PSD | 0.07 | 0.01 | 118 | 4.47 | <0.001* |
| Scaler: Normaliser | -0.01 | 0.02 | 118 | -0.66 | 0.51 |
| Scaler: Robust | 0.01 | 0.02 | 118 | 0.51 | 0.61 |
| Scaler: Standard | 0.01 | 0.02 | 118 | 0.40 | 0.69 |
| ModelType: MLP+PCA | -0.02 | 0.02 | 118 | -1.36 | 0.18 |
| ModelType: XGBoost | 0.03 | 0.02 | 118 | 1.64 | 0.10 |
| ModelType: XGBoost+PCA | -0.01 | 0.02 | 118 | -0.88 | 0.38 |
| Classification of *Ae. aegypti, An. stephensi,* and *Cx. quinquefasciatus* | | | | | |
| Variable | Estimate | SE | df | t.ratio | p.value |
| (Intercept) | 0.65 | 0.08 | 118 | 7.75 | <0.001* |
| Feature: MFCC | 0.16 | 0.03 | 118 | 6.58 | <0.001* |
| Feature: PSD | 0.07 | 0.03 | 118 | 2.68 | <0.01* |
| Scaler: Normaliser | 0.02 | 0.03 | 118 | 0.62 | 0.53 |
| Scaler: Robust | 0.02 | 0.03 | 118 | 0.58 | 0.56 |
| Scaler: Standard | 0.02 | 0.03 | 118 | 0.58 | 0.56 |
| ModelType: MLP+PCA | -0.03 | 0.03 | 118 | -0.97 | 0.34 |
| ModelType: XGBoost | 0.03 | 0.03 | 118 | 1.15 | 0.25 |
| ModelType: XGBoost+PCA | -0.04 | 0.03 | 118 | -1.32 | 0.19 |

**S4 Table**. **Pair-wise comparisons of model inputs for the tested classification algorithms**. Asterisk (*) indicates p-value smaller than 0.05 (p<0.05).

| Classification of *Ae. aegypti*, *Ae. albopictus*, and *Cx. quinquefasciatus* | | | | | |
| --- | --- | --- | --- | --- | --- |
| Comparison | Estimate | SE | df | t.ratio | p.value |
| Fundamental- MFCC | -0.12 | 0.01 | 118 | -8.29 | <0.001* |
| Fundamental- PSD | -0.07 | 0.01 | 118 | -4.48 | <0.001* |
| MFCC-PSD | 0.05 | 0.01 | 118 | 3.77 | <0.001* |
| No Scaler- Normaliser | 0.01 | 0.02 | 118 | 0.66 | 0.91 |
| No Scaler- Robust Scaler | -0.01 | 0.02 | 118 | -0.52 | 0.96 |
| No Scaler- Standard Scaler | -0.01 | 0.02 | 118 | -0.40 | 0.98 |
| Normaliser- Robust Scaler | -0.02 | 0.02 | 118 | -1.24 | 0.60 |
| Normaliser- Standard Scaler | -0.02 | 0.02 | 118 | -1.12 | 0.68 |
| Robust Scaler- Standard Scaler | 0.00 | 0.02 | 118 | 0.13 | 1.00 |
| MLP- MLP+PCA | 0.02 | 0.02 | 118 | 1.36 | 0.53 |
| MLP- XGBoost | -0.03 | 0.02 | 118 | -1.64 | 0.36 |
| MLP- XGBoost+PCA | 0.01 | 0.02 | 118 | 0.88 | 0.81 |
| MLP+PCA- XGBoost | -0.05 | 0.02 | 118 | -3.17 | 0.01* |
| MLP+PCA- XGBoost+PCA | -0.01 | 0.02 | 118 | -0.54 | 0.95 |
| XGBoost- XGBoost+PCA | 0.04 | 0.02 | 118 | 2.77 | 0.03* |
| Classification of *Ae. aegypti, An. stephensi,* and *Cx. quinquefasciatus* | | | | | |
| Comparison | Estimate | SE | df | t.ratio | p.value |
| Fundamental- MFCC | -0.16 | 0.02 | 118 | -6.60 | <.0001* |
| Fundamental- PSD | -0.07 | 0.03 | 118 | -2.69 | 0.02* |
| MFCC- PSD | 0.10 | 0.02 | 118 | 3.91 | <0.001* |
| No Scaler- Normaliser | -0.02 | 0.03 | 118 | -0.63 | 0.92 |
| No Scaler- Robust Scaler | -0.02 | 0.03 | 118 | -0.59 | 0.94 |
| No Scaler- Standard Scaler | -0.02 | 0.03 | 118 | -0.59 | 0.94 |
| Normaliser- Robust Scaler | 0.00 | 0.03 | 118 | 0.07 | 1.00 |
| Normaliser- Standard Scaler | 0.00 | 0.03 | 118 | 0.07 | 1.00 |
| Robust Scaler- Standard Scaler | 0.00 | 0.03 | 118 | 0.00 | 1.00 |
| MLP- MLP+PCA | 0.03 | 0.03 | 118 | 0.97 | 0.77 |
| MLP- XGBoost | -0.03 | 0.03 | 118 | -1.15 | 0.66 |
| MLP- XGBoost+PCA | 0.04 | 0.03 | 118 | 1.32 | 0.55 |
| MLP+PCA- XGBoost | -0.06 | 0.03 | 118 | -2.24 | 0.12 |
| MLP+PCA- XGBoost+PCA | 0.01 | 0.03 | 118 | 0.36 | 0.98 |
| XGBoost- XGBoost+PCA | 0.07 | 0.03 | 118 | 2.66 | 0.04* |
